# Caregiver adherence to outpatient follow-up of children infected with or exposed to syphilis during pregnancy

**DOI:** 10.1016/j.jped.2025.101493

**Published:** 2025-12-26

**Authors:** Márcia Galdino Sampaio, Cristina Barroso Hofer

**Affiliations:** a Hospital Federal dos Servidores do Estado, Rio de Janeiro, RJ, Brazil; b Universidade Federal do Rio de Janeiro (UFRJ), Curso de Doenças Infecto-Parasitárias, Rio de Janeiro, RJ, Brazil; c Universidade Federal do Rio de Janeiro (UFRJ), Departamento de Doença**s** Infecto-Parasitárias, Rio de Janeiro, RJ, Brazil; d Universidade Federal do Rio de Janeiro (UFRJ), Programa de Pós-graduação em Saúde Materno-Infantil, Rio de Janeiro, RJ, Brazil

**Keywords:** Congenital syphilis, Treatment adherence, Clinical protocol, Syphilis

## Abstract

**Objective:**

To describe the caregiver adherence to the various interventions proposed by the Ministry of Health in the follow-up of children infected with or exposed to *Treponema pallidum* during pregnancy.

**Method:**

This is a prospective cohort study that included 256 children treated for congenital syphilis during the neonatal period. The children were referred from maternity to a reference outpatient clinic in the state of Rio de Janeiro between 2016 and 2021. Adherence was used as the outcome variable. It was assessed in two components: clinical-laboratory adherence (basic adherence) and adherence to specialist consultations (final adherence). Factors associated with adherence were investigated using logistic regression.

**Results:**

It was observed that 41 % of the children were followed for at least 18 months, and 68 % had two consecutive non-reactive VDRL tests. Basic adherence was 32 %, while final adherence (including specialist consultations and clinical-laboratory follow-up) was only 16 %. Additionally, 36 children presented permanent sequelae during follow-up. Factors such as higher maternal age (OR = 1.10; 95 % CI:1.03–1.20) and the presence of permanent sequelae in the children (OR = 4.87; 95 % CI: 2.29–10.35) were predictors of adherence. Loss to follow-up occurred in 68 % (173/256) of the cases.

**Conclusion:**

This study highlights a very low level of caregiver adherence to the congenital syphilis management protocol recommended by the Brazilian Ministry of Health. The presence of sequelae in children and higher maternal age were associated with improved adherence.

## Introduction

Congenital syphilis remains highly prevalent in Brazil and is associated with significant morbidity and mortality [1]. Most infected newborns are asymptomatic, yet serious sequelae may occur even after adequate treatment, underscoring the need for follow-up during early childhood [2,3]. Monitoring enables the detection of abnormalities and timely interventions during a critical period of development [4]. Late congenital syphilis results from untreated early lesions or persistent inflammation, predominantly affecting bones, teeth, and the central nervous system [5].

Diagnosis remains challenging because no single test reliably confirms or excludes infection, and most serological tests detect maternal IgG transferred across the placenta [6]. Given the above, the Brazilian Ministry of Health (MS) and several international organizations recommend follow-up of children exposed to or infected with Treponema pallidum during pregnancy for at least 18 months [7]. During follow-up, several interventions are proposed, with minimal differences between protocols [8, 9, 10, 11]. In Brazil, clinical and laboratory follow-up is recommended for the child, with two consecutive non-reactive VDRL tests before the sixth month of life [7]. Since 2019, children have been referred to primary care services for serological and clinical monitoring and, when necessary, are referred by pediatric care to specialist evaluation, [4] representing a shift from earlier protocols that emphasized routine specialist assessment for all exposed infants. This change is particularly relevant in settings with limited specialist availability, as it reduces unnecessary referrals of clinically normal exposed infants.

Evidence indicates that adherence to long-term care remains low even among patients with chronic health conditions [12]. This challenge is likely to be even greater among asymptomatic children who have already completed treatment for congenital syphilis in maternity services, reinforcing the need to identify groups at higher risk of loss to follow-up and to develop more effective monitoring strategies.

Although several studies have examined the incidence and initial management of congenital syphilis, few have investigated long-term adherence to outpatient follow-up. This gap is particularly relevant in middle-income countries like Brazil, where social determinants and structural inequalities may hinder continuity of care [13,14].

The primary objective of this study was to evaluate adherence to the follow-up interventions recommended by the Ministry of Health among children exposed to or reported for congenital syphilis. The secondary objective was to identify maternal and neonatal factors associated with loss to follow-up.

## Methods

This is a prospective cohort study conducted between 2016 and 2021, involving children referred from maternity hospitals to the outpatient clinic of a public hospital specializing in sexually transmitted infections (STIs) in the state of Rio de Janeiro. All referred children who attended the outpatient clinic had their consultations scheduled and were followed by a pediatrician. However, only those meeting the eligibility criteria were included in the study. The inclusion criteria were: (1) exposure to syphilis during pregnancy (at birth, they did not meet the criteria for congenital syphilis notification, meaning no clinical, laboratory, or imaging findings compatible with congenital syphilis, and their mothers were adequately treated during pregnancy; (2) children meeting the criteria for congenital syphilis notification (newborns whose mothers were untreated or inadequately treated but who showed no clinical, laboratory, or imaging signs compatible with congenital syphilis); (3) children with congenital syphilis (defined as the presence of clinical, laboratory, or imaging findings compatible with syphilis and/or a neonatal VDRL titer greater than two dilutions above the maternal VDRL titer at birth).

Exclusion criteria were: (1) age over six months at the first consultation; (2) children with genetic disorders or congenital malformations; and (3) children treated for syphilis after the first month of life.

According to the guidelines of MS, outpatient follow-up was conducted until 18 to 24 months of age by pediatricians and specialists. Three specialist evaluations were recommended – ophthalmology, audiology, and neurology – to be performed between six and 18 to 24 months of age. Children were referred to these specialists through a formal consultation request, and the findings were recorded in the medical chart.

Adherence to follow-up was defined as:

- Clinical and laboratory adherence (basic adherence): clinical follow-up until at least 18 months of age and two consecutive non-reactive VDRL tests (With the second performed after three months of age)
- Adherence to specialists: the child attended at least one consultation with a neurologist, an ophthalmologist, and an audiologist.
- Final adherence: adherence to both clinical and laboratory follow-up and adherence to specialist consultations.
- Loss to follow-up: the child did not complete clinical follow-up for at least 18 months and did not achieve two consecutive non-reactive VDRL results.
- Permanent abnormalities: neurological, auditory, or ophthalmologic conditions diagnosed by specialists that persisted until the end of follow-up.

In addition, the authors defined small for gestational age (SGA) neonates as those born below the 10th percentile for gestational age.

All data were collected in a database built in Excel (Office 365) and subsequently exported and analyzed using the Statistical Package for Social Science (SPSS®), version 21.0–2012 for Windows. Continuous variables were graphically assessed for distribution and described using mean, minimum, and maximum values. Categorical variables were initially described using absolute numbers and percentages. For comparisons, adherence definitions were used as the outcome variable. The Student’s *t*-test was applied to compare the means of continuous independent variables, and Fisher’s exact test was used for categorical variables. Factors associated with adherence were analyzed using binary logistic regression. Variables with *p* < 0,25 in the bivariate analysis were included in the multivariate model, with selection performed using the backward method. The model fit was assessed using the Hosmer-Lemeshow test [15]. All hypotheses were two-tailed, with a significance level set at 0,05. Variables with less than 5 % missing data were analyzed using complete-case analysis, whereas those exceeding this threshold were excluded to avoid bias.

The study was approved by the Research Ethics Committee of Instituto de Puericultura e Pediatria Martagão Gesteira (IPPMG) at the Federal University of the State of Rio de Janeiro: CAAE: 77,073,017.0.0000.5264 and the General Hospital of Nova Iguaçu (HGNI): CAAE: 77,073,017.0.3002.8044.

## Results

A total of 256 children were included, of whom 14 % (35/256) were born symptomatic, and 26 % (67/256) met notification criteria, despite 94.5 % (242/256) of mothers having attended prenatal care. Additionally, 75 % of pregnant women attended an adequate number of consultations (six or more), but 32 % (82/256) were not treated, and in 5 % (9/174), treatment was inadequate. Maternal age ranged from 13 to 44 years (mean 23.6 years). Notably, 99 % (254/256) of newborns received some treatment regimen for syphilis during hospitalization.

The mean age at the first follow-up visit was 2.4 months, and 68 % achieved two consecutive non-reactive VDRL results, with a mean age of 7.8 months at the second test. The treponemal tests performed at the end of follow-up were non-reactive (69/256) (Table 1).

**Table 1:** Laboratory follow-up of children exposed to or diagnosed with congenital syphilis at the STI Outpatient Clinic (RJ), 2016–2021.

Numerical Variables	Descriptive Statistics	
	N ( %)	Mean (months)-(Min-Max)^a^
First VDRL performed (frequency and mean age)	247 (96.5)	3.7 (1–18)
Second VDRL performed (frequency and mean age)	190 (74.2)	7.1 (2–27)
Two non-reactive VDRL (frequency and mean age)	174 (68)	7.8 (3–27)
Treponemal test (frequency and mean age)	69 (27)	20.2 (5–39)

A total of 39 % (100/256) of children attended at least one consultation with all specialists, totaling 684 evaluations, but adherence declined nearly 90 % between the first and last visits (Table 2). Overall, 36 children (14 %) presented 40 permanent abnormalities (six auditory, 25 neurological, nine ophthalmological). In the analysis of basic adherence, prenatal care, illicit drug use, and small-for-gestational-age status were significant in the bivariate model but not after adjustment. Maternal age, when analyzed as a continuous variable, remained associated with adherence, but this association was lost after categorization (Table 3). Only permanent alterations remained independently associated (crude OR = 7.43; 95 %CI: 3.37–16.37; aOR = 6.71; 95 %CI: 2.76–16.32).

**Table 2:** Specialist follow-up of children exposed to or diagnosed with congenital syphilis at the STI Outpatient Clinic (RJ), 2016–2021.

Specialist consultations	First consultation N ( %)/ Mean (months, min–max)^a^	Second consultation N ( %) / Mean (months, min–max)	Third consultation N ( %) / Mean (months, min–max)
Neurology	142 (55.5) / 9.8 (1–63)	66 (25.8) / 15.7 (5–33)	22 (8.6) / 20.6 (13–43)
Ophthalmology	143 (55.8) / 10.4 (1–38)	45 (17.6) / 16.4 (5–39)	7 (2.7) / 24.2 (16–35)
Speech therapy / Audiology	161 (62.0) / 6.6 (1–22)	77 (30.0) / 13.6 (4–43)	21 (8.2) / 18.1 (7–29)

**Table 3:** Simple and multivariate logistic regression of maternal sociodemographic factors, prenatal care, maternal behavioral factors, child birth conditions, and outpatient follow-up associated with basic adherence. STI Clinic (RJ), 2016–2021.

Variables *N* = 256	Basic adherence^a^		p value	Crude OR^b^ (95 % CI^c^)	Adjusted OR (95 % CI^c^)
	No N ( %)	Yes N ( %)			
Prenatal care attendance *N* = 242	168 (69)	74 (31)	0.02	0.24 (0.08–0.75)	1.01 (0.17–5.92)
Tobacco use during pregnancy *N* = 253	26 (59)	18 (41)	0.21	1.60 (0.82–3.13)	1.01 (0.39–2.58)
Illicit drug use during pregnancy *N* = 253	7 (39)	11 (61)	< 0.01	3.70 (1.37–9.94)	1.63 (0.33–7.94)
Alcohol use during pregnancy *N* = 253	66 (68)	31 (32)	1.00	0.99 (0.57–1.71)	
History of miscarriage *N* = 251	23 (57,5)	17(42,5)	0.13	1.77 (0.88–3.55)	1.58 (0.72–3.48)
History of stillbirth *N* = 251	13 (81)	3 (19)	0.40	0.48 (0.13–1.74)	
Employment status *N* = 256	32 (76)	10 (24)	0.21	0.60 (0.28–1.29)	0.51 (0.21–1.25)
Maternal age category *N* = 253			0.10		1.77 (0.92–3.41)
< 20 years	45 (76)	14 (24)			
20–34 years	122 (67)	61 (33)			
> 34 years	5 (45)	6 (55)			
Maternal age (mean in years) *N* = 253	22.9	25.16	0.03	1.07 (1.02–1.13)	1.08 (1.02–1.14)
Maternal education (mean in years) *N* = 251	9.77	9.71	0.85	0.99 (0.89–1.09)	
Adequate treatment during prenatal care *N* = 256	112 (68)	53 (32)	0.90	1.03 (0.60–1.79)	
Family income (mean in BRL) (*N* = 254)	1.244,72	1.071,69	0.07	1.00 (0.99–1.00)	1.00 (0.99–1.00)
Number of living children *N* = 253			0.29	1.46 (0.75–2.84)	
Up to two	144 (70)	63 (30)			
More than two	28 (61)	18 (39)			
Prematurity *N* = 256	8 (57)	6 (43)	0.39	1.60 (0.53–4.79)	
Low birth weight *N* = 255	9 (47)	10 (53)	0.07	2.48 (0.96–6.36)	0.52 (0.11–2.28)
Small for gestational age (SGA) *N* = 255	7 (39)	11 (61)	0.01	3.60 (1.34–9.66)	3.11 (0.80–12.07)
Child classification *N* = 256			1.00	0.99 (0.58–1.69)	
Exposed *N* = 154	104 (67.5)	50 (32.5)			
Infected + Notified *N* = 102	69 (68)	33 (32)			
children with permanent alterations *N* = 256	10	26	<0001	7.43 (3.37–16.37)	6.71 (2.76–16.32)

a Basic adherence (clinical and laboratory adherence) - clinical follow-up until at least 18 months of age and two consecutive non-reactive VDRL tests.

b OR, Odds ratio; c- CI, Confidence Interval.

For final adherence (42/256), higher maternal age and permanent alterations were the main predictors (aOR = 1.10; 95 %CI: 1.03–1.17; aOR = 6.37; 95 %CI: 2.60–15.59) (Table 4).

**Table 4:** Simple and multivariate logistic regression of maternal sociodemographic variables, prenatal care, maternal behavioral factors, child birth conditions, and outpatient follow-up associated with the outcomes of having final adherence. STI clinic (RJ), 2016–2021.

Variables *N* = 256	Final adherence^a^		p value	Crude OR^b^ (95 %CI)	Adjusted OR (95 %CI^c^)
	No N ( %)	Yes N ( %)			
Prenatal care attendance *N* = 242	203 (84)	39 (16)	0.70	0.70 (0.18–2.64)	
Tobacco use during pregnancy *N* = 253	36 (82)	8 (18)	0.65	1.18 (0.50–2.77)	
Illicit drug use during pregnancy *N* = 253	12 (67)	6 (33)	0.049	2.85 (1.00–8.11)	3.67 (0.81–16.50)
Alcohol use during pregnancy *N* = 253	82 (85)	15 (15)	0.86	0.91 (0.45–1.82)	
History of miscarriage *N* = 251	31 (77.5)	9 (22,5)	0.24	1.62 (0.70–3.73)	1.26 (0.48–3.30)
History of stillbirth *N* = 251	14 (87.5)	2 (12.5)	1.00	0.71 (0.15–3.28)	
History of syphilis treatment in the past *N* = 256	35 (80)	9 (20)	0.50	1.39 (0.61–3.17)	
Employment status *N* = 256	36 (86)	6 (14)	0,82	0.82 (0.32–2.10)	
Maternal age (mean in years) *N* = 253	23.13	26.1	<0.01	1.09 (1.03–1.16)	1.10 (1.03–1.17)
Maternal age category (*N* = 253)			0.003		2.52 (1.09–5.84)
< 20 years	59 (89)	7 (11)			
20–34 years	148 (83)	30 (17)			
> 34 years	4 (44)	5 (56)			
Maternal education (mean in years) *N* = 251	9.80	9.49	0.69	0.97 (0.85–1.10)	
Adequate treatment during prenatal care *N* = 256	138 (84)	27 (16)	1.00	1.00 (0.50–2.01)	
Family income (mean in BRL) *N* = 254	1.232,75	964.02	0.02	0.99 (0.99–1.00)	0.99 (0.99–1.00)
Number of living children *N* = 253			1.00	0.91 (0.37–2.21)	
Up to two	173 (84)	34 (16)			
More than two	39 (85)	7 (15)			
Prematurity *N* = 256	11 (79)	3 (21)	0.70	1.42 (0.37–5.32)	
Low birth weight *N* = 255	13 (68)	6 (32)	0.09	2,56 (0.91–7.18)	1.03 (0.26–4.01)
Small for gestational age (SGA) *N* = 255	15 (83)	3 (17)	1.00	1.01 (0.28–3.67)	
Child classification *N* = 256			1.00	1,03 (0.52–2.02)	
Exposed	129 (84)	25 (16)			
Infected + Notified	85 (83)	17 (17)			
children with permanent alterations *N* = 256	20	16	<0.001	5.96 (2.75–12.94)	6.37 (2.60–15.59)

a Final adherence was defined as being followed for at least 18 months, achieving two consecutive non-reactive VDRL tests, and attending at least one consultation with a neurologist, an ophthalmologist, and an audiologist.

b OR, Odds ratio; c-CI, Confidence Interval.

No collinearity was detected among the independent variables (tolerance 0.61–0.91; VIF < 2), and the Hosmer–Lemeshow test indicated a good model fit for both analyses (basic follow-up: χ² = 9.31, *p* = 0.317; final adherence: χ² = 6.8, *p* = 0.549).

## Discussion

The follow-up of children exposed to or infected with *Treponema pallidum* during pregnancy is essential for identifying late manifestations and sequelae and ensuring timely interventions [13].

In this study, which evaluates adherence to follow-up of children exposed to or infected with *Treponema pallidum* during pregnancy, the authors observed 32 % adherence to basic follow-up and 16 % to total follow-up, considering visits to specialists. However, classic socioeconomic inequities, such as non-White skin color, low income, fewer years of schooling, lack of prenatal care, and inadequate treatment during pregnancy, were not statistically associated with adherence to follow-up. Regarding the basic adherence component, the apparent protective effect of prenatal care observed in the crude model was explained by confounding: younger mothers were more likely to attend prenatal care but exhibited lower adherence overall. Additionally, in the Brazilian context, attendance alone does not guarantee high-quality prenatal care, particularly for women with syphilis. National assessments, such as the study by Tomasi et al., [16] demonstrate substantial heterogeneity and social inequality in prenatal care quality, with essential components of comprehensive prenatal care frequently omitted, including adequate counseling, risk assessment, routine laboratory testing, and continuity of follow-up.

Although illicit drug use showed no independent association with adherence, unmeasured confounding may explain this finding. In many cases, children of mothers with substance use were brought by other caregivers, potentially masking the direct effect of maternal behavior. This highlights the role of support networks in contexts of social vulnerability.

Higher maternal age remained independently associated with adherence. When maternal age was categorized, more than one quarter of mothers in the non-adherent group were adolescents, compared with a smaller proportion among those who adhered. This finding aligns with the well-described vulnerabilities of adolescent motherhood, such as lower autonomy, reduced health literacy, unstable support networks, and greater difficulty navigating health services [17].

A study conducted at the Federal University of Paraná Hospital [13] found that maternal age over 30 years was associated with discontinuation of follow-up, whereas in our cohort, higher maternal age, both as a continuous and categorical variable, was significantly associated with greater final adherence. Similar findings were reported in a prospective cohort study conducted in disadvantaged communities in England, [18] where increasing maternal age was associated with better physical health, improved language development, and higher vaccination adherence in children up to five years of age when compared with children of younger mothers. Together, these results suggest that maternal maturity and experience may enhance understanding of the importance of follow-up care and engagement with health services. Conversely, younger mothers may face more socioeconomic and emotional barriers that hinder adherence, reinforcing the need for additional counseling and social support strategies for this group.

Several inadequacies in the follow-up recommended by the MS were observed in this cohort. The mean age at the first consultation was two months, while the recommendation is that the first visit should occur within the first month of life [7]. The same phenomenon was observed in a retrospective study conducted in three public maternity hospitals and an infectious disease service in a city in Minas Gerais, which identified 54 children meeting the criteria for congenital syphilis. Of these, 79,6 % were not even referred for outpatient follow-up, [19] demonstrating difficulties in complying with actions recommended by the MS. Kawaguchi et al. reported outpatient follow-up records for children treated for syphilis in maternity hospitals up to six months of age in only 9.9 % of cases. [20] Similarly, Lago et al., [21] in a cohort of 398 children, showed that 30 % of those who met the criteria for syphilis and 22.5 % of those exposed were followed for only eight months of life. In the current study, both the group that met the criteria for syphilis and the exposed group demonstrated a basic adherence rate of 32 %, with a mean follow-up duration of 16 months. One possible explanation for the lack of adherence is that most children are asymptomatic at birth [7] and receive treatment in the maternity hospital, demonstrating that outpatient follow-up until serological negativization is very challenging in patients who have already shown clinical improvement. During follow-up, the Brazilian Ministry of Health recommends 10 to 12 appointments until the child reaches 18 to 24 months of age [7]. Despite follow-up appointments being scheduled by the pediatrician within the same facility, the number of consultations was lower than recommended, with an average of six per patient.

An exploratory and descriptive study conducted in primary care in Fortaleza, Ceará, analyzed the child healthcare (puericulture) of children whose mothers had gestational syphilis [22] found that none underwent VDRL testing during routine visits, indicating major gaps in follow-up. In contrast, laboratory adherence was the best-performing indicator in our cohort: 68 % achieved two consecutive non-reactive VDRL tests. The mean age of seroreversion was 7.8 months, later than the 3.8 months reported in another study, [14] although 86 % achieved their first non-reactive result between one and three months of age. These findings reinforce the need for team training, improved network organization, and streamlined workflows to ensure timely testing, especially because the absence of seroreversion after six months requires reassessment [7].

The treponemal test was performed in only 27 % of the children, all of whom were non-reactive. It was observed that 34 % of the children who met criteria for infection at birth achieved seroreversion. One study [21] found that among 550 children who met the notification criteria for syphilis (born to mothers without adequate treatment) but were asymptomatic, only two had a reactive treponemal test after 18 months. Conversely, only 10 % of the children with infection criteria achieved seroreversion after 18 months. It is suggested that the treponemal test be performed at the end of follow-up (after 18 months) to detect IgG antibodies [7]. A reactive result at this age indicates that the child had syphilis, as maternal antibodies would no longer be present. However, a non-reactive result after 18 months does not rule out infection at birth. Studies show that children with clinical signs at birth consistent with syphilis may still achieve seroreversion during follow-up [21,23]. A study conducted in Canada [24] with 16 children meeting the criteria for congenital syphilis showed that 11 (68.8 %) had a negative treponemal test after 18 months, while five (31.2 %) did not achieve seroreversion. Among these five, 60 % received treatment starting after the second month of life. It is believed that treatment initiated in utero or immediately after birth may influence the humoral response in the context of an immature immune system [11,24]. The São Paulo Society of Pediatrics recommends that infants with a reactive treponemal test after 18 months should undergo long-term follow-up, at least until the age of five [23].

Another important factor contributing to poor adherence, as reported in the literature, is financial hardship [13]. Although mean family income was not statistically associated with adherence in our analysis, this likely reflects the socioeconomic homogeneity of the study population, composed predominantly of low-income families receiving care through the public health system. The municipality where the study was conducted presents marked social vulnerability, which limits variability in income and may obscure statistical associations. Nonetheless, structural barriers related to poverty, such as transportation difficulties and limited access to services, continue to indirectly affect follow-up. Therefore, in order to improve adherence, it is essential that follow-up care be provided at primary care facilities close to the family’s residence. This approach allows for monitoring missed appointments, conducting active searches, and developing strategies to improve the engagement of both the children and their caregivers in the follow-up process.

Starting in 2019, the Ministry of Health limited specialist referrals to children with clinical alterations [7]. However, specialized care in Brazil continues to face major constraints, with high demand and long waiting lists. In our cohort, specialist follow-up showed substantial losses, with over a 90 % reduction in subsequent evaluations despite all visits being pre-scheduled. This suggests that factors other than appointment availability may further influence continuity of specialist care. In contrast, primary care offers a comprehensive set of recommended follow-up actions that, when properly implemented, allow early detection of problems. The Pan American Health Organization (PAHO) International Seminar on Specialized Health Care emphasizes strengthening primary care by expanding coverage, increasing problem-solving capacity, and improving access management [25]. Key child-health actions include recording anthropometric data in the vaccination card using World Health Organization (WHO) growth charts and assessing neurodevelopment across four domains: personal-social, fine motor-adaptive, gross motor, and language development [26].

A cohort study reported sequelae in 13.3 % of children exposed to or treated for congenital syphilis; in our study, 14 % presented permanent conditions. Caregivers of these children were six times more likely to adhere to follow-up, likely reflecting greater concern when health needs are evident. In contrast, a qualitative study showed that non-adherence often stems from caregivers seeking care only when the child is symptomatic, highlighting the prioritization of curative rather than preventive actions [27]. The lower adherence among asymptomatic children highlights the persistent challenge of health systems that prioritize curative over preventive care. When no symptoms are present, health professionals may fail to prioritize asymptomatic children, and families may likewise underestimate the need for follow-up, reinforcing a well-recognized barrier to continuity of preventive services.

A limitation of this study is that maternal data were obtained from prenatal care booklets and maternity discharge summaries, which may contain recording errors or underreporting. Additionally, the study included two pandemic years, which could have affected attendance. Although our internal comparison found no significant differences in adherence between pandemic and non-pandemic periods, this remains a limitation and reflects pre-existing weaknesses in care. These similar adherence patterns suggest that longstanding gaps in access, continuity, and caregiver support were already influencing follow-up for children exposed to congenital syphilis.

This cohort shows low caregiver adherence to the follow-up recommended by the Ministry of Health. Permanent child conditions and higher maternal age were associated with greater adherence, while younger mothers showed lower continuity of care. Evidence indicates that low adherence is largely driven by structural health-system barriers, and our findings reflect the same pattern. Strengthening Primary Health Care, improving coordination between maternity services and primary care, and implementing active tracking, especially for infants of younger mothers, may help reduce losses to follow-up. Policies that expand access, continuity of care, and support for vulnerable families are essential to ensure consistent follow-up and improve outcomes.

## Data availability statement

The data that support the findings of this study are available from the corresponding author.

## Conflicts of interest

The authors declare no conflicts of interest.

## References

[1] Ministério da Saúde (BR) (2024). Boletim epidemiológico de sífilis.

[2] Kollmann T.R., Dobson S.S., Remington J.S., Klein J.O. (2011). Infectious diseases of the fetus and newborn infant.

[3] Ministério da Saúde (BR) (2006). Diretrizes para controle da sífilis congênita: manual de bolso.

[4] Ministério da Saúde (BR) (2019). *Protocolo* clínico e diretrizes terapêuticas para prevenção da transmissão vertical do HIV, sífilis e hepatites virais.

[5] Hollier L.M., Cox S.M. (1998). Syphilis. Semin Perinatol..

[6] Herremans T., Kortbeek L., Notermans D.W. (2010). A review of diagnostic tests for congenital syphilis in newborns. Eur J Clin Microbiol Infect Dis.

[7] Ministério da Saúde (BR) (2022). Sífilis e hepatites virais.

[8] Workowski K.A., Bachmann L.H., Chan P.A., Johnston C.M., Muzny C.A., Park I. (2021). Sexually transmitted infections treatment guidelines, 2021. MMWR Recomm Rep.

[9] Fanella S., Bitnun A., Sauve L. (2024). Diagnosis and management of congenital syphilis: avoiding missed opportunities. Paediatr Child Health.

[10] Janier M., Unemo M., Dupin N., Tiplica G.S., Potočnik M., Patel R. (2021). European guideline on the management of syphilis. J Eur Acad Dermatol Venereol.

[11] Dai Y., Zhai G., Zhang S., Chen C., Li Z., Shi W. (2022). The clinical characteristics and serological outcomes of infants with confirmed or suspected congenital syphilis in Shanghai, China: a hospital-based study. Front Pediatr.

[12] Tavares N.U., Bertoldi A.D., Mengue S.S., Arrais P.S., Luiza V.L., Oliveira M.A. (2016). Factors associated with low adherence to medicine treatment for chronic diseases in Brazil. Rev Saude Publica.

[13] Feliz M.C., Prizybicien A.R., Rossoni A.M., Tahnus T., Pereira A.M., Rodrigues C. (2016). Adherence to the follow-up of the newborn exposed to syphilis and factors associated with loss to follow-up. Rev Bras Epidemiol.

[14] Cavalcante A.N., Araujo M.A., Nobre M.A., Almeida R.L. (2019). Factors associated with inadequate follow-up of children with congenital syphilis. Rev Saude Publica.

[15] Bursac Z., Gauss C.H., Williams D.K., Hosmer D.W. (2008). Purposeful selection of variables in logistic regression. Source Code Biol Med.

[16] Tomasi E., Fernandes P.A., Fischer T., Siqueira F.C., da Silveira D.S., Thumé E. (2017). Quality of prenatal services in primary healthcare in Brazil: indicators and social inequalities. Cad Saude Publica.

[17] Silva S.S., Santos M.S., Estevam K.D., Magalhães J.G., Beserra C.R., Macena F.C. (2024). Gravidez na adolescência no Brasil: determinantes sociais, culturais e econômicos. Braz J Implant Health Sci.

[18] Sutcliffe A.G., Barnes J., Belsky J., Gardiner J., Melhuish E. (2012). The health and development of children born to older mothers in the United Kingdom: observational study using longitudinal cohort data. BMJ.

[19] Lafetá K.R., Martelli H., Silveira M.F., Paranaíba L.M (2016). Maternal and congenital syphilis, underreported and difficult to control. Rev Bras Epidemiol.

[20] Kawaguchi I.A.L., Magalhães D.M.S., Calderon I.M.P., Dias A.A. (2014). O seguimento de sífilis congênita em crianças tratadas ao nascer. Com Cienc Saude.

[21] Lago E.G., Vaccari A., Fiori R.M. (2013). Clinical features and follow-up of congenital syphilis. Sex Transm Dis.

[22] Oliveira F.A., Araujo M.A., Barros V.L., Guanabara M.A., Vasconcelos L.D., Sena M.V. (2023). Childcare and follow-up of children exposed to syphilis or notified with congenital syphilis. Texto Contexto Enferm.

[23] Sociedade Paulista de Pediatria, Pediatra Atualize-se (2017). https://www.spsp.org.br/site/asp/boletins/AT08.pdf.

[24] Singh A.E., Guenette T., Gratrix J., Bergman J., Parker P., Anderson B. (2013). Seroreversion of treponemal tests in infants meeting Canadian surveillance criteria for confirmed early congenital syphilis. Pediatr Infect Dis J.

[25] Brasil. Ministério da Saúde (2023). https://redeaps.org.br/informativos-e-noticias/noticias/seminario-internacional-de-atencao-especializada-no-sus/12410/.

[26] Brasil. Ministério da Saúde (2012). Departamento de atenção básica. saúde da criança: crescimento e desenvolvimento.

[27] Godeiro A.L., Gurgel P.K., Santos A.D., Monteiro A.I. (2013). Participation in monitoring child development: how is the adherence of caregivers?. Rev APS.

